# Liuweibuqi capsules improve pulmonary function in stable chronic obstructive pulmonary disease with lung-qi deficiency syndrome by regulating STAT4/STAT6 and MMP-9/TIMP-1

**DOI:** 10.1080/13880209.2019.1666151

**Published:** 2019-11-03

**Authors:** Dan-Dan Shen, Zhong-Hui Yang, Ji Huang, Fei Yang, Zi-Wei Lin, Ying-Fei Ou, Min-Hao Hu

**Affiliations:** a Department of Pharmacy, The First People’s Hospital of Taicang, Affiliated Hospital of Soochow University, Taicang, P. R. China;; b Office of the Party and Government, The First People’s Hospital of Taicang, Affiliated Hospital of Soochow University, Taicang, P. R. China;; c Nanjing University of Chinese Medicine, Nanjing, P. R. China;; d Department of Rehabilitation Medicine, The First People’s Hospital of Taicang, Affiliated Hospital of Soochow University, Taicang, P. R. China

**Keywords:** IFN-γ, IL-6, IL-4, signal transducer and activator of transcription, forced expiratory volume, forced vital capacity, matrix metalloproteinase

## Abstract

**Context:** Liuweibuqi (LWBQ) capsule has been reported to influence symptoms of patients with chronic obstructive pulmonary disease (COPD); however, specific function of LWBQ capsules in COPD with lung-qi deficiency syndrome remains elusive.

**Objective:** This study investigates effect of LWBQ capsules on STAT4/STAT6 and MMP-9/TIMP-1 expression and pulmonary function in stable COPD with lung-qi deficiency syndrome.

**Materials and methods:** Totally, 429 patients diagnosed with stable COPD and lung-qi deficiency syndrome were treated with starch capsules (each time for 9 capsules), or different doses: low (each dose for 8 capsules and 1 LWBQ capsules), medium (each time for 6 capsules and 3 LWBQ capsules), or high (each time for 9 LWBQ capsules) of LWBQ capsules for 30 days, 3 times a day. Forced expiratory volume in 1 s (FEV1), forced vital capacity (FVC), FEV1/FVC% and DLco%pred were evaluated by pulmonary function meter. STAT4/STAT6 and MMP-9/TIMP-1 expression was assessed by RT-qPCR and western blot analysis, and serum concentrations of IL-4, IFN-γ and IL-6 by ELISA.

**Results:** Spearman rank correlation analysis and ROC curve showed that STAT4/STAT6 and MMP-9/TIMP-1 affected pulmonary functions and curative effect of stable COPD with lung-qi deficiency syndrome. After LWBQ capsule treatment, FEV1, FVC, FEV1/FVC% and DLco%pred elevated; STAT4/STAT6, MMP-9/TIMP-1, IFN-γ and IL-6 expression declined whereas IL-4 expression increased (*p* < 0.05). Logistic regression analysis demonstrated that FEV1/FVC was negatively correlated with STAT4/STAT6 and MMP-9/TIMP-1 expression in COPD patients.

**Conclusions:** LWBQ capsules play a beneficial role in pulmonary function of stable COPD with lung-qi deficiency syndrome *via* STAT4/STAT6 and MMP-9/TIMP-1.

## Introduction

Chronic obstructive pulmonary disease (COPD) is an obstructive lung disease characterized by pulmonary dysfunction and persistent airflow limitation, which is usually related with an elevated chronic inflammatory response of the lungs (Li et al. 2016). COPD ranked third among the main causes accounting for morbidity and mortality around the world in 2020 (Raherison 2011). COPD has been associated with an elevated risk of contracting multiple comorbidities. Smoking is considered as the most significant contributor in COPD but other factors such as exposure to biological agents can also promote its exacerbation (Barreiro and Criner 2014). People with stable COPD always present with symptoms including emphysema, pulmonary hypertension, small airway remodeling, and chronic bronchitis (Li et al. 2012). In accordance with evidence-based medicine, inhaled glucocorticosteroids, short-acting bronchodilators, long-acting bronchodilators, and low-dose, slow-release theophylline are the well-established therapeutic treatment regimens for COPD patients (Li et al. 2012). However, despite medical treatment, the prognosis and recovery of many COPD patients is still unfavorable (Wang et al. 2017a). Therefore, superior medications and more effective strategies are urgently needed for treating patients with COPD.

Liuweibuqi (LWBQ) capsules are a type of Traditional Chinese Medicine (TCM), which comprise of Renshen, Yuzhu, Huangqi, Rougui, Yizhi, and Chenpi (Wang et al. 2017a). Previous research found that LWBQ capsules can reduce the inflammatory reaction, thereby improving the symptoms of COPD by modulating the expression of matrix metalloproteinase-9 (MMP-9) and tissue inhibitor of matrix metalloproteinase-1 (TIMP-1) (Wang et al. 2015). MMP’s are prominent mediators in the turnover of extracellular matrix (ECM), which is maintained by the balanced expression between the pro- and anti-proteolytic factors including MMP-9 and its specific inhibitor-TIMP-1 (Barr et al. 2010). The signal transducer and activator of transcription (STAT) proteins, which are composed of a family of cytoplasmic transcription factors, critically function by modulating a variety of biological processes (Page et al. 2013). Previous studies have demonstrated the vital roles of both STAT4 and STAT6 in immune responses against inflammation in patients with inflammatory bowel diseases and in Alternaria-induced asthma mice (Kim et al. 2012; Wu et al. 2016). In the present study, a total of 429 patients diagnosed as stable COPD with lung-qi deficiency syndrome were selected and treated with different doses of LWBQ capsules. Then the effect of LWBQ capsules on stable COPD with lung-qi deficiency syndrome and its underlying mechanism with STAT4/STAT6 and MMP-9/TIMP-1 were illustrated.

## Materials and methods

### Ethics statement

Our experiments were performed in accordance with the ethical standards formulated in the Helsinki Declaration. All the patients signed informed consents prior to the study. Our experiments were approved by the ethics committee of the First People’s Hospital of Taicang, Soochow University, Affiliated Hospital of Soochow University (Clinical Trial Registration Number: ChiCTR1900025511).

### Study subjects

From June 2011 to October 2015, a total of 429 patients diagnosed with stable COPD and lung-qi deficiency syndrome were selected for this study from the Respiratory Department of the First People’s Hospital of Taicang, Soochow University, Affiliated Hospital of Soochow University. The patients were randomly classified into 4 groups using the random grouping system RandA1.0. The COPD groups were assigned into 3 groups according to the treatment regimens: low dose of LWBQ (low-dose) group with 103 cases, medium dose of LWBQ (medium-dose) group with 100 cases, high dose of LWBQ (high-dose) group with 105 cases and the remaining 121 cases were regarded as control. Stable COPD was verified by lung volume assessment (FEV1/FVC < 70%) according to the current guidelines defined by the Global Initiative for Chronic Obstructive Lung Disease (GOLD). The lung qi deficiency syndrome followed the diagnostic standard as defined in the guidelines for the Clinical Research of Chinese Medicine New Drugs formulated by the State Food and Drug Administration. The inclusion criteria for the patients were as follows: patients confirmed as stable COPD with lung-qi deficiency syndrome; patients aging from 30 to 70; patients without abnormalities of the other systems. No restrictions were defined for parameters such as gender, body temperature, body weight, smoking, and GOLD classification (no significant difference was found performed by paired *t*-test, *p* > 0.05). The exclusion criteria were as follows: patients diagnosed with other primary pulmonary diseases; patients diagnosed with diseases of the other systems; COPD patients with other syndromes except for lung-qi deficiency (Kawayama et al. 2016).

### Drug treatment

Patients in each group were subjected to routine treatment according to the state of illness (Gentry & Gentry 2017). 0: patients were treated with influenza vaccination to avoid the risk factors; I: patients were added with short-acting bronchodilators as needed; II: on the basis of I stage treatment, patients were added with long-acting bronchodilator one or more times regularly and treated with pulmonary rehabilitation; III: on the basis of II stage treatment, patients were treated with glucocorticoid (nasal route) if pathogenetic condition exacerbated; IV: on the basis of III stage treatment, if chronic respiratory failure was diagnosed, selective operation was conducted. The patients in each group were treated according to the following treatment regimens for 30 days: routine treatment + capsules (starch inside and designed to have similar taste and smell as LWBQ capsules) (3 times a day and each time for 9 capsules), routine treatment + low dose of LWBQ (3 times a day and each time for 8 capsules and 1 LWBQ capsules), routine treatment + medium dose of LWBQ (3 times a day and each time for 6 capsules and 3 LWBQ capsules) and routine treatment + high dose of LWBQ (3 times a day and each time for 9 LWBQ capsules). LWBQ capsules were produced by the Pharmaceutical Center of the First Affiliated Hospital of Anhui University of Traditional Chinese Medicine (Hefei, Anhui, China), whose ingredients included 10 g sun-dried ginseng, 5 g Huangqi, 6 g Alpinia Oxyphylla, 10 g Yuzhu, 3 g Rougui and 6 g Chenpi.

### Evaluation of pulmonary function

A technician explained and checked the detailed operation. The COSMED pulmonary function meter (PONY FX, Shanghai Sanliwen Medical Equipment Co. Ltd, Shanghai, China) was used to detect the forced expiratory volume in 1 s (FEV1) and the forced vital capacity (FVC) of patients. Then the ratio of FEV1/FVC (%) and the predicted value of carbon monoxide diffusion capacity (DLco%pred) were calculated. The detection was repeated 3 times and the optimal value was selected.

### Reverse transcription quantitative polymerase chain reaction (RT-qPCR)

Endobronchial biopsies were performed in COPD patients and the normal volunteers for lung tissue samples (Dong et al. 2016). The total RNA was extracted from the samples using an ultra-pure RNA extraction kit (D203-01, Beijing GenStar Biosolutions Co. Ltd, Beijing, China). The primers of STAT4, STAT6, MMP-9, TIMP-1 and glyceraldehyde-3-phosphate dehydrogenase (GAPDH) were designed and synthesized by TaKaRa Co. Ltd, (Tokyo, Japan) (Table 1). The RNA template, Primer Mix, dNTP Mix, DTT, RT Buffer, HiFi-MMLV and anhydrous RNase were dissolved over ice for the following experiment. Reverse transcription (20 μL) was performed according to the instructions of the TaqMan MicroRNA Assays Reverse Transcription Primer kit (4366596, thermo scientific, Waltham, MA, USA). The reaction condition was as follows: reverse transcription at 42 °C for 30–50 min and reverse transcriptase enzyme inactivation at 85 °C for 5 s. The reaction liquid was used for fluorescent quantitation PCR according to the SYBR® Premix Ex TaqTM II reagent kit (RR820A, Action-award Biotech, Co. Ltd. Guangzhou, Guangdong Province, China). The reaction system was as follows: 25 µL SYBR® Premix Ex TaqTM II (2×), 2 µL forward primer, 2 µL reverse primer, l μL ROX Reference Dye (50×), 4 µL DNA template, and 16 µL double distilled water (ddH_2_O). Fluorescent quantitation PCR was conducted using the ABI PRISM® 7300 system (Prism®7300, Shanghai Kunke Instrumenttation Co. Ltd, Shanghai, China). The reaction condition was as follows: pre-denaturation at 95 °C for 10 min, a total of 40 cycles of denaturation at 95 °C for 15 s and annealing at 60 °C for 30 s, then extension at 72 °C for 1 min. GAPDH was served as an internal reference. The relative expression of the target gene was calculated based on the 2^−ΔΔCt^ method, in which ΔΔCt = (the mean Ct value of the target gene in the COPD group) – the mean Ct value of the house-keeping gene in the COPD group) – (the mean Ct value of the target gene in the control group) – the mean Ct value of the house-keeping gene in the control group). The experiment was repeated three times.

**Table 1.** The primer sequence of STAT4, STAT6, MMP-9, TIMP-1 and GAPDH.

Targeted genes	Sequences	Tm°C
STAT4	F: GCTTAACAGCCTCGATTTCAAGA	60
	R: GAGCATGGTGTTCATTAACAGGT	61
STAT6	F: GTTCCGCCACTTGCCAATG	62
	R: TGGATCTCCCCTACTCGGTG	62
MMP-9	F: TGTACCGCTATGGTTACACTCG	61
	R: GGCAGGGACAGTTGCTTCT	60
TIMP-1	F: ACCACCTTATACCAGCGTTATGA	58
	R: GGTGTAGACGAACCGGATGTC	59
GAPDH	F: GGAGCGAGATCCCTCCAAAAT	55
	R: GGCTGTTGTCATACTTCTCATGG	60

STAT4: signal transducers and activators of transcription 4; STAT6: signal transducers and activators of transcription 6; MMP-9: matrix metalloproteinase-9; TIMP-1: tissue inhibitor of metalloproteinases-1; GAPDH: glyceraldehyde-3-phosphate dehydrogenase; F: forward; R: reverse.

### Western blot analysis

Gradient centrifugation was conducted in order to isolate the peripheral blood mononuclear cells (PBMC) using Ficoll-hypaque (GE Healthcare, Munich, USA). Then the cells were added with Radio-Immunoprecipitation Assay (RIPA) lysis buffer: 50 mM Tris pH 7.5, 150 mM NaCl, 1％Triton X-100, 1 mM EGTA, 1% sodium deoxycholate and phosphatase, 1% protease inhibitor cocktail (Sigma-Aldrich Co., St. Louis, MO, USA) and 0.5 mM sodium orthovanadate. The mixture was then transferred into the centrifuge tube and incubated on ice for 30 min, and then shaken every 10 min. After centrifugation (12,000 rpm) at 4 °C for 30 min, the lipid layer was removed. The protein concentration was analyzed using a bicinchoninic acid (BCA) protein assay kit (20201ES76, Shanghai Yeasen Biotech, Co. Ltd, Shanghai, China). Protein sample (30 μg) adjusted by deionized water was mixed with the loading buffer, boiled at 100 °C for 5 min, incubated in ice, centrifuged and separated by electrophoresis using 10% SDS separating gel and stacking gel. Then the protein was transferred from the gel onto a nitrocellulose membrane. The membrane was blocked with 5% skimmed milk overnight at 4 °C with diluted rabbit monoclonal anti STAT4 (ab68156, 1:1000, Abcam, USA), monoclonal anti STAT6 (ab32520, 1:1000, Abcam, USA), polyclonal anti MMP-9 (ab73734, 100 µg 0.9 mg/mL, Abcam, USA) and polyclonal anti TIMP-1 (ab211926, 100 µL 2.279 mg/mL, Abcam, USA). After three washes with phosphate buffered solution (PBS) for 5 min each, the membrane was incubated with horseradish peroxidase (HRP)-conjugated goat anti-rabbit IgG secondary antibody (ab6721, Abcam, USA) at 37 °C for 1 h and then washed again with PBS 3 times for 5 min each. The membrane was soaked in the chemiluminescence (ECL) reagent (Pierce, Waltham, MA, USA) for 1 min at room temperature and then covered with preservative film after removal of excessive liquid. Then the membrane was exposed to X-rays and imaged in conditions devoid of light. GAPDH served as the internal reference. The ratio of gray value between the target protein band and internal reference band was considered as the relative expression of protein.

### Enzyme linked immunosorbent assay (ELISA)

The detection was performed in accordance with the instructions of human interleukin (IL)-4 ELISA Kit (ab100570, Abcam, Shanghai), human IL-6 ELISA Kit (ab178013, Abcam, Shanghai) and human interferon (IFN) gamma ELISA Kit (ab174443, Abcam, Shanghai). Each well was added with 100 μL standard diluent, 100 μL IL-4 (with the concentration of 320, 160, 80, 40, 20 and 0 pg/mL), the samples (diluted at 1:10) and Biotinylated anti-human IL-4 solution. Then the mixture was stirred for 3 h at room temperature, after which the residual liquid was removed and the plate was washed four times. Next, the plate was incubated for 30 min at room temperature with 100 μL horseradish peroxidase (HRP)-conjugated anti-rabbit IgG antibody and the blank well was set as control. Subsequently, the residual liquid was removed and the plate was rewashed four times, followed by the addition of 100 μL stable chromogen into each well including the blank well. When the solution returned to blue, the plate was incubated at room temperature in conditions devoid of light. The development was terminated by addition of 50 μL stop buffer. Optical density (OD) at 450 nm was measured within 20 min. The detection of IL-6 and IFN-γ was conducted in the same manner following the above steps. IL-6 Protein Human Lyophilized Recombinant Protein and IFNG Human Lyophilized Recombinant Protein were added to the wells before shaking.

### Evaluation of curative effect

The curative effect was assessed according to the *Guidelines for the Clinical Research of Chinese Medicine New Drugs.* Effectiveness: The symptoms of cough, phlegm, asthma and chest tightness improved; lung rale reduced; the integral quantity was reduced by more than 40%; the pulmonary function and self-care ability improved. Ineffectiveness: no change in the symptoms of cough, phlegm, asthma, chest tightness and lung rale; the integral quantity was reduced by less than 40%; the pulmonary function and self-care ability decreased.

### Follow-up

After 6 months, the patients went back to the hospital to re-measure the content of FEV1, FVC, FEV1/FVC%, DLco%pred, IFN-γ, IL-6 and IL-4. The detected data of 4 groups were involved in the analysis of results.

### Statistical analysis

Statistical analyses were conducted using the SPSS 21.0 software (IBM, Armonk, NY, USA). Measurement data were expressed as mean ± standard deviation. Differences between groups before and after treatment were compared by paired *t-*test, and differences among multiple groups were compared by one-way ANOVA, followed by Tukey's *post hoc* test. The enumeration data were expressed as percentage or decimal and analyzed by Spearman rank correlation analysis. The diagnostic value of STAT4, STAT6, MMP-9 and TIMP-1 on patients diagnosed as stable COPD with lung-qi deficiency syndrome was analyzed by receiver operating characteristic (ROC) curve. A value of *p* < 0.05 was considered to be significant.

## Results

### All enrolled patients meet the inclusion criteria

A total of 429 patients were randomly classified into 4 groups using the random grouping system RandA1.0. No significant differences were observed in the baseline characteristics (*p* > 0.05) in terms of parameters such as age, gender, body mass index (BMI), smoking, and GOLD classification after the analysis of *t*-test (Table 2). Prior to the study, the patients were confirmed without any other systemic diseases, no family history of other diseases, no infectious diseases or drug allergy history, which may affect our results.

**Table 2.** Baseline characteristics for included patients.

Baseline characteristics	Control group	Low-dose group	Medium-dose group	High-dose group	*p* Value
	(*n* = 121)	(*n* = 103)	(*n* = 100)	(*n* = 105)	
Age	56.85 ± 4.67	58.21 ± 5.04	57.95 ± 4.76	57.56 ± 4.72	0.162
Gender (male/female)	68/53	56/47	54/46	55/50	0.953
BMI (kg/m^2^)	20.79 ± 1.61	20.62 ± 1.68	20.76 ± 1.50	20.74 ± 1.84	0.886
body temperature (°C)	37.05 ± 0.49	36.80 ± 0.49	36.84 ± 0.58	36.84 ± 0.53	0.656
Smoking history					
Often [*n* (%)]	17 (14.05%)	13 (12.62%)	11 (11.00%)	14 (13.33%)	0.283
Never [*n* (%)]	43 (35.54%)	38 (36.89%)	34 (34.00%)	51 (48.57%)	
Ever [*n* (%)]	61 (50.41%)	52 (50.49%)	55 (55.00%)	40 (38.10%)	
GOLD classification					
The first stage [*n* (%)]	9 (7.44%)	7 (6.80%)	14 (14.00%)	9 (8.57%)	0.356
The second stage [*n* (%)]	76 (62.81%)	59 (57.28%)	48 (48.00%)	53 (50.48%)	
The third stage [*n* (%)]	28 (23.14%)	30 (29.13%)	27 (27.00%)	31 (29.52%)	
The fourth stage [*n* (%)]	8 (6.61%)	7 (6.80%)	11 (11.00%)	12 (11.43%)	

BMI: body mass index; GOLD: Global Initiative for Chronic Obstructive Lung Disease.

### LWBQ capsules can improve the pulmonary function

The COSMED pulmonary function meter was utilized to detect the pulmonary function. Before the treatment, no differences were evident for the contents of FEV1, FVC, FEV1/FVC% and DLco%pred in each group (*p* > 0.05). After treatment and follow-up visits, the contents of FEV1, FVC, FEV1/FVC% and DLco%pred in the COPD groups elevated in comparison with the control group; the contents of FEV1, FVC, FEV1/FVC% and DLco%pred in medium-dose and high-dose groups elevated in comparison with the low-dose group; the contents of FEV1, FVC, FEV1/FVC% and DLco%pred in high-dose groups elevated in comparison with the medium-dose group (all *p* < 0.05) (Table 3). LWBQ capsules could effectively restore the lung function in COPD patients, with variable therapeutic effect in a dose-dependent manner.

**Table 3.** LWBQ capsules can improve the pulmonary function indicated by pulmonary function tests.

Pulmonary function index	Before treatment				After treatment				Follow-up			
	Control group	Low-dose group	Medium-dose group	High-dose group	Control group	Low-dose group	Medium-dose group	High-dose group	Control group	Low-dose group	Medium-dose group	High-dose group
FEV1	1.02 ± 0.27	1.03 ± 0.30	0.99 ± 0.24	1.01 ± 0.28	1.16 ± 0.34*	1.38 ± 0.34*#	1.68 ± 0.36*#△	1.83 ± 0.39*#▲	1.54 ± 0.32*	1.78 ± 0.37*#	2.08 ± 0.36*#△	2.36 ± 0.37*#▲
FVC	1.81 ± 0.47	1.79 ± 0.46	1.78 ± 0.47	1.80 ± 0.48	1.96 ± 0.51*	2.14 ± 0.51*#	2.34 ± 0.51*#△	2.55 ± 0.54*#▲	2.21 ± 0.46*	2.39 ± 0.46*#	2.59 ± 0.47*#△	2.77 ± 0.47*#▲
FEV1/FVC(%)	56.99 ± 6.50	56.95 ± 6.44	56.08 ± 5.43	56.33 ± 6.80	59.05 ± 8.42*	64.08 ± 6.75*#	71.89 ± 4.42*#△	74.53 ± 5.82*#▲	69.97 ± 6.78*	74.51 ± 4.88*#	80.60 ± 5.22*#△	85.65 ± 5.11*#▲
DLco%pred	61.07 ± 6.00	61.5 ± 5.78	61.67 ± 5.40	61.54 ± 5.89	65.31 ± 6.44*	71.93 ± 6.88*#	77.42 ± 7.41*#△	82.96 ± 8.23*#▲	73.12 ± 6.94*	77.38 ± 6.56*#	82.62 ± 6.37*#△	87.43 ± 6.24*#▲

*, *p* < 0.05, *vs.* the detection results before treatment; #, *p* < 0.05, *vs.* the control group; △, *p* < 0.05, *vs.* the low-dose group; ▲, *p* < 0.05, *vs.* the medium-dose group; FEV1: forced expiratory volume in 1 s; FVC: forced vital capacity; DLco%pred: the predicted value of carbon monoxide diffusing capacity.

### LWBQ capsules can down-regulate STAT4/STAT6 and MMP-9/TIMP-1

**Figure 1.**
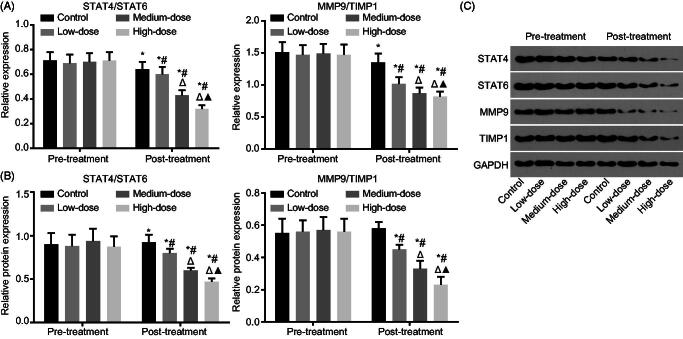
LWBQ capsules contribute to reduce expression of STAT4/STAT6 and MMP-9/TIMP-1. (A) Relative mRNA expression of STAT4/STAT6 and MMP-9/TIMP-1 were decreased by conducting RT-qPCR; (B and C) Relative protein levels of STAT4/STAT6 and MMP-9/TIMP-1 were decreased by conducting western blot analysis; *, *p* < 0.05, *vs.* the detection results before treatment; #, *p* < 0.05, *vs.* the control group; △, *p* < 0.05, *vs.* the low-dose group; ▲, *p* < 0.05, *vs.* the medium-dose group; STAT4: signal transducers and activators of transcription 4; STAT6: signal transducers and activators of transcription 6; MMP-9: matrix metalloproteinase-9; TIMP-1: tissue inhibitor of metalloproteinases-1; GAPDH: glyceraldehyde-3-phosphate dehydrogenase; RT-qPCR: reverse transcription quantitative polymerase chain reaction.

The expression of STAT4, MMP-9, STAT6 and TIMP-1 was measured by RT-qPCR and western blot analysis. The results (Figure 1) presented that the utilization of LWWQ would repress the expression of STAT4/STAT6 and MMP-9/TIMP-1 (all *p* < 0.05). Before treatment, no significant differences were detected in regard to the expression of STAT4/STAT6 and MMP-9/TIMP-1 (*p* > 0.05). However, after treatment, the expression of STAT4/STAT6 and MMP-9/TIMP-1 was decreased in all groups compared with the expression detected prior to the treatment (*p* < 0.05). In comparison with the expression exhibited in the control group, the expression of STAT4/STAT6 and MMP-9/TIMP-1 was decreased in the COPD groups; the expression of STAT4/STAT6 and MMP-9/TIMP-1 was decreased in medium-dose and high-dose groups compared with the expression in the low-dose group; the expression of STAT4/STAT6 and MMP-9/TIMP-1 was decreased in high-dose groups compared with the expression in the medium dose group (all *p* < 0.05). These results showed that LWBQ capsules decreased the expression of STAT4/STAT6 and MMP9/TIMP1 in a dose-dependent manner. In addition, another key observation was that the LWBQ capsules could affect the expression of STAT4/STAT6 and MMP9/TIMP1 in COPD patients.

### LWBQ capsules decrease COPD-mediated inflammation

**Figure 2.**
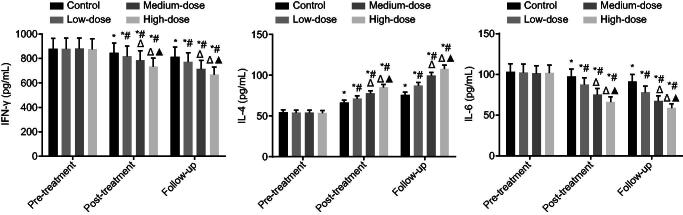
LWBQ capsules repress the expression of inflammatory cytokines. #, *p* < 0.05, *vs.* the control group; △, *p* < 0.05, *vs.* the low-dose group; ▲, *p* < 0.05, *vs.* the medium-dose group; IFN-γ: interferon-γ; IL-6: interleukin-6; IL-4: interleukin-4.

The serum concentrations of IL-4, IFN-γ and IL-6 were evaluated by ELISA. The utilization of LWBQ would reduce the expression of IFN-γ and IL-6 while inducing the expression of IL-4 (all *p* < 0.05). Before treatment, no significant differences were detected in regard to the expression of IL-4, IFN-γ and IL-6 (*p* > 0.05). After treatment and in follow-up visits, the expression of IFN-γ and IL-6 decreased, while the expression of IL-4 increased in all groups compared with the expression before treatment (*p* < 0.05). In comparison with the control group, the expression of IFN-γ and IL-6 decreased, and the expression of IL-4 increased in the COPD groups. The expression of IFN-γ and IL-6 decreased and the expression of IL-4 increased in the medium-dose and high-dose groups compared with the expression detected in the low-dose group. The expression of IFN-γ and IL-6 was decreased; the expression of IL-4 was increased in the high-dose groups compared with the expression in the medium dose group (all *p* < 0.05) (Figure 2). These results showed that LWBQ capsule decreased the secretion of pro-inflammatory cytokines IFN-γ and IL-6, and increased the secretion of anti-inflammatory cytokine IL-4 in a dose-dependent manner. These results provide evidence supporting the ability of LWBQ capsules to inhibit lung inflammation in COPD patients.

### The balances of STAT4/STAT6 and MMP-9/TIMP-1 are closely linked with pulmonary function

Spearman rank correlation analysis was employed to analyze the correlation among FEV1, FVC, FEV1/FVC%, DLco%pred, MMP-9/TIMP-1 and STAT4/STAT6. The results demonstrated that FEV1 was positively correlated with FVC, FEV1/FVC% and DLco%pred (all *p* < 0.05, *r* = 0.91, *r* = 0.59, *r* = 0.38). FVC was positively correlated with FEV1/FVC% and DLco%pred (*p* < 0.05, *r* = 0.23, *r* = 0.21). FEV1/FVC% was negatively correlated with MMP-9/TIMP-1 and STAT4/STAT6 (*p* < 0.05, *r* = −0.62, *r* = −0.55). MMP-9/TIMP-1 was positively correlated with STAT4/STAT6 (*p* < 0.05, *r* = −0.71) (Table 4). These results suggested that the expression of MMP9/TIMP1 and STAT4/STAT6 was related to an improved recovery of lung function in COPD patients after treatment with LWBQ capsules.

**Table 4.** Pulmonary function was correlated with the expression of STAT4/STAT6 and MMP-9/TIMP-1.

Indicators	FEV1	FVC	FEV1/FVC%	DLco%pred	MMP-9/TIMP-1	STAT4/STAT6
FEV1		0.91	0.59	0.38	−0.55	−0.49
FVC	0.91*		0.23	0.21	−0.35	−0.3
FEV1/FVC%	0.59*	0.23*		0.48	−0.62	−0.55
DLco%pred	0.38*	0.21*	0.48*		−0.61	−0.51
MMP-9/TIMP-1	−0.55*	−0.35*	−0.62*	−0.61*		0.71
STAT4/STAT6	−0.49*	−0.30*	−0.55*	−0.51*	0.71*	

FEV1: forced expiratory volume in 1 s; FVC: forced vital capacity; DLco%pred: the predicted value of carbon monoxide diffusing capacity; STAT4: signal transducers and activators of transcription 4; STAT6: signal transducers and activators of transcription 6; MMP-9: matrix metalloproteinase-9; TIMP-1: tissue inhibitor of metalloproteinases-1.

### STAT4/STAT6 and MMP-9/TIMP-1 may serve as diagnostic indicators for patients in stable COPD with lung-qi deficiency syndrome

**Figure 3.**
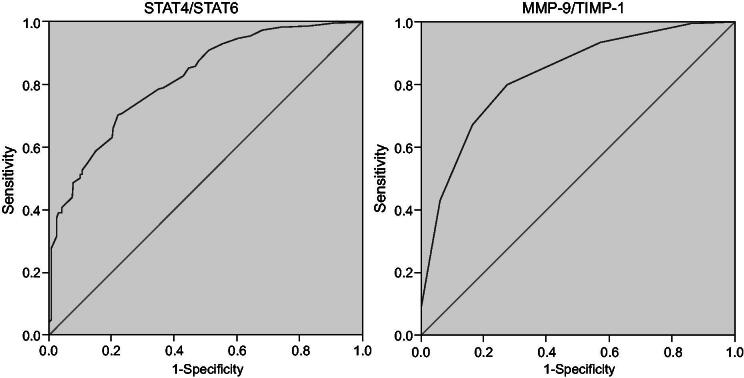
STAT4, STAT6, MMP-9 and TIMP-1 are potential biomarkers for stable COPD with lung-qi deficiency syndrome. STAT4: signal transducers and activators of transcription 4; STAT6: signal transducers and activators of transcription 6; MMP-9: matrix metalloproteinase-9; TIMP-1: tissue inhibitor of metalloproteinases-1.

The diagnostic value of STAT4, STAT6, MMP-9 and TIMP-1 in patients diagnosed as stable COPD with lung-qi deficiency syndrome was depicted by the ROC curve. For STAT4/STAT6, the area under ROC curve was 0.817 with a critical value of 0.815, sensitivity of 0.701 and specificity of 0.783. For MMP-9/TIMP-1, the area under ROC curve was 0.822 with a critical value of 0.175, sensitivity of 0.795 and specificity of 0.726 (Figure 3). The findings suggested that the diagnostic value of STAT4, STAT6, MMP-9 and TIMP-1 in stable COPD with lung-qi deficiency syndrome was in the middle level. These results further demonstrated that LWBQ capsules could inhibit the expression of STAT4/STAT6 and MMP-9/TIMP-1 and control the pulmonary inflammatory response in COPD patients. Besides, it also suggested the therapeutic use of STAT4/STAT6 and MMP-9/TIMP-1 as prognostic indicators for COPD treated by LWBQ capsules.

### Pulmonary function, STAT4/STAT6 and MMP-9/TIMP-1 were all influencing factors of curative effect of LWBQ capsules in treating patients with COPD

Logistic regression analysis was conducted to identify the influencing factors for the curative effect with effect as dependent variable, STAT4/STAT6 (critical value: 0.815), MMP-9/TIMP-1 (critical value: 0.175) and pulmonary function indexes (critical value: 0.7) as independent variables. Pulmonary functions and the expression of STAT4/STAT6 and MMP-9/TIMP-1 would influence the curative effect of stable COPD with lung-qi deficiency syndrome (all *p* < 0.05) (Table 5). Logistic regression analysis showed that FEV1/FVC was negatively correlated with STAT4/STAT6 and MMP-9/TIMP-1 expression in COPD patients.

**Table 5.** Pulmonary function indexes, expressions of STAT4/STAT6, MMP-9/TIMP-1 all affect curative effect.

	B	S.E	Wald value	*p*	EXP(B)	EXP(B)95%CI	
						Lower limit	Upper limit
FEV1/FVC%	−7.544	1.051	51.526	< 0.001	0.001	0	0.004
STAT4/STAT6	3.104	0.81	14.674	< 0.001	22.29	4.554	109.111
MMP-9/TIMP-1	5.005	0.922	29.484	< 0.001	149.129	24.491	908.079

FEV1: forced expiratory volume in 1 s; FVC: forced vital capacity; STAT4: signal transducers and activators of transcription 4; STAT6: signal transducers and activators of transcription 6; MMP-9: matrix metalloproteinase-9; TIMP-1: tissue inhibitor of metalloproteinases-1.

## Discussion

COPD is a common cause of mortality related to lung disease, and it has been anticipated to be the third most leading cause of mortality by the year 2020 (Celli et al. 2012). TCM has been integrated in the course of COPD treatment for a long time, which led to the discovery of its beneficial effects on suppressing the frequency of acute exacerbations and improving the patients’ quality of life (Li et al. 2012). Therefore, in our study, we evaluated the effect of one TCM, LWBQ capsules, in stable COPD patients with lung-qi deficiency syndrome. Then we disclosed that the LWBQ capsules could improve pulmonary function in patients of stable COPD with lung qi deficiency by up-regulating the expression of STAT6 and TIMP-1, down-regulating the expression of STAT4 and MMP-9.

In our study, we preliminary observed that after treatment with LWBQ capsules, the pulmonary function indexes FEV1, FVC, FEV1/FVC% and DLco%pred increased in different degree with significantly increased numbers in patients treated with a high dose of LWBQ capsules. FEV1/FVC is commonly used to define the degree of an airway obstruction by the Global Initiative for Chronic Obstructive Lung Disease (GOLD), and DLco%pred is an indicator of postoperative complications in arterial oxygen desaturation during exercise and thoracic surgery in the COPD patients (Osuka et al. 2015). In this study, the results suggested that LWBQ capsules could effectively restore the lung function of COPD patients, and variable therapeutic effect in a dose-dependent manner. Besides, the logistic regression analysis also showed a negative correlation of FEV1/FVC with STAT4/STAT6 and MMP-9/TIMP-1 expression in COPD patients.

Our findings suggested that LWBQ capsules could reduce the expression of STAT4/STAT6 and MMP-9/TIMP-1 in patients suffering from stable COPD with lung-qi deficiency syndrome. Especially upon treatment with a higher dose of LWBQ capsules, the balanced expression of STAT4/STAT6 and MMP-9/TIMP-1 could be inevitably maintained, which aided in the improvement of COPD symptoms with other medications. The research of Huang et al. (2010) stated that treatment with astragalosides decreased MMP-9 expression while it increased TIMP-1 expression in mice with ischemia-reperfusion injury. Functioning as a crucial facilitator in an inflammatory reaction, MMP-9 inhibition could amplify the effectiveness of a treatment regimen in COPD and asthma through its regulation on airway modeling (Grzela et al. 2016). A vital link has been demonstrated between MMP-9 and emphysema; moreover, elevated MMP-9 expression levels are related to a decreased diffusing capacity and further severe airflow obstruction, thereby providing crucial evidence regarding the involvement of MMP-9 in the pathogenesis of COPD (Chaudhuri et al. 2013). Yao et al. (2013) elucidated a declined expression of TIMP-1, with an enhanced expression of MMP-9 in the lungs of patients with COPD as well as in mouse lungs with emphysema, proving theoretical basis supporting that only TIMP-1/MMP-9 balance could protect against COPD/emphysema. By conducting a meta-analysis, Li et al. (2016) indicated that the imbalance of MMP-9/TIMP-1 could have a relation with COPD pathogenesis, suggesting that the MMP-9 and TIMP-1 proteins could act as a paramount biological indicator in the diagnosis of COPD. A research demonstrated the involvement of STAT4 in the development of cigarette smoke-induced small airway remodeling in mice lungs, and stated that the aberrant expression of STAT4 regulates the matrix production and airway fibroblast phenotype, thereby providing a promising target for human COPD treatment (Hackett et al. 2014). The results of a previous investigation presented that STAT4 was up-regulated in smokers with COPD while STAT6 was down-regulated in asthmatic bronchial epithelium, which was consistent with our results (Di Stefano et al. 2004; Mullings et al. 2001). The current study conducted an investigation of inflammatory response upon LWBQ capsule treatment, which presented that LWBQ capsules can repress the inflammatory response in patients by decreasing the expression of pro-inflammatory cytokines-IFN-γ and IL-6 along with increasing the expression of IL-4, which was coincident with a previous study (Wang et al. 2017a). The involvement of overexpressed IL-4 has been reported in glucocorticoid resistance of different chronic Th2 diseases featured with fibrotic remodeling or impaired apoptotic cell clearance, including COPD, and IFN-γ production is associated with glucocorticoid-resistant asthma (Zizzo & Cohen 2013). These aforementioned are supportive of the ability of LWBQ capsules to inhibit lung inflammation in COPD patients.

Oral Huangqi, an ingredient of LWBQ capsules as well as a primary herb for strengthening Qi, exercises its beneficial effects in lung function improvement, life quality, and also in decreasing the incidence of exacerbations for patients with stable COPD (Wu et al. 2013). An et al. (2011) proved that Ginseng formulae (the ingredient of LWBQ capsules) also showed promising effects on lung functions along with an improved quality of life for patients with stable COPD. In addition, Chenpi (the ingredient of LWBQ capsules) has been reported to protect against wheezing, dyspnea, and cough, and consequently relieving asthma, a respiratory disease, using a cough guinea pig model induced by citric acid and an experimental asthma guinea pig model induced by histamine (Shi et al. 2009). A clinical study manifested that over-exposure to various exogenous pathogenic factors, which further causes impairment of lung qi and lung deficiency as well as yin-yang disharmony, accounts for the increasing incidence of COPD, while TCM therapy for COPD always conforms to the principles of eliminating the causative agent and enhancing the bodily constitution (Dong et al. 2014). Furthermore, Wang and colleagues (2017b) attested that LWBQ capsules could mitigate the progression of COPD by inhibiting the inflammatory response upon regulation of the JAK/STAT pathway in a COPD rat model.

## Conclusions

In summary, the current clinical study suggests that LWBQ capsules could reduce inflammatory responses, improve pulmonary function of people suffering from stable COPD with lung-qi deficiency by up-regulating the expression of STAT6 and TIMP-1, down-regulating the expression of STAT4 and MMP-9. Hence, our study speculates LWBQ capsules as a novel therapeutic drug for stable COPD with lung-qi deficiency. Though our study reveals that LWBQ capsules are effective in COPD treatment, we also acknowledge that the approval for these capsules in a clinical setting still waits. Therefore, further experiments with a larger sample size are needed for more accurate data and to verify the creditability of these results. Moreover, the side-effect of LWBQ capsules should be investigated in the future.

## References

[CIT0001] An X, Zhang AL, Yang AW, Lin L, Wu D, Guo X, Shergis JL, Thien FC, Worsnop CJ, Xue CC. 2011. Oral ginseng formulae for stable chronic obstructive pulmonary disease: a systematic review. Respir Med. 105(2):165–176. 21146973 10.1016/j.rmed.2010.11.007

[CIT0002] Barr TL, Latour LL, Lee KY, Schaewe TJ, Luby M, Chang GS, El-Zammar Z, Alam S, Hallenbeck JM, Kidwell CS, Warach S. 2010. Blood-brain barrier disruption in humans is independently associated with increased matrix metalloproteinase-9. Stroke. 41(3):e123–e128. 20035078 10.1161/STROKEAHA.109.570515 PMC2827673

[CIT0003] Barreiro E, Criner GJ. 2014. Update in chronic obstructive pulmonary disease 2013. Am J Respir Crit Care Med. 189(11):1337–1344. 24881938 10.1164/rccm.201402-0245UP

[CIT0004] Celli BR, Locantore N, Yates J, Tal-Singer R, Miller BE, Bakke P, Calverley P, Coxson H, Crim C, Edwards LD, et al. 2012. Inflammatory biomarkers improve clinical prediction of mortality in chronic obstructive pulmonary disease. Am J Respir Crit Care Med. 185(10):1065–1072. 22427534 10.1164/rccm.201110-1792OC

[CIT0005] Chaudhuri R, McSharry C, Spears M, Brady J, Grierson C, Messow CM, Miele G, Nocka K, MacNee W, Connell M, et al. 2013. Sputum matrix metalloproteinase-9 is associated with the degree of emphysema on computed tomography in COPD. Transl Respir Med. 1(1):11–11. 27234393 10.1186/2213-0802-1-11 PMC6733425

[CIT0006] Di Stefano A, Caramori G, Capelli A, Gnemmi I, Ricciardolo FL, Oates T, Donner CF, Chung KF, Barnes PJ, Adcock IM. 2004. STAT4 activation in smokers and patients with chronic obstructive pulmonary disease. Eur Respir J. 24(1):78–85. 15293608 10.1183/09031936.04.00080303

[CIT0007] Dong L, Xia JW, Gong Y, Chen Z, Yang HH, Zhang J, He J, Chen XD. 2014. Effect of lianhuaqingwen capsules on airway inflammation in patients with acute exacerbation of chronic obstructive pulmonary disease. Evid Based Complement Alternat Med. 2014:637969. 24971150 10.1155/2014/637969 PMC4058171

[CIT0008] Dong R, Xie L, Zhao K, Zhang Q, Zhou M, He P. 2016. Cigarette smoke-induced lung inflammation in COPD mediated via LTB4/BLT1/SOCS1 pathway. Int J Chron Obstruct Pulmon Dis. 11:31–41. 26730186 10.2147/COPD.S96412 PMC4694688

[CIT0009] Gentry S, Gentry B. 2017. Chronic obstructive pulmonary disease: diagnosis and management. Am Fam Physician. 95(7):433–441. 28409593

[CIT0010] Grzela K, Litwiniuk M, Zagorska W, Grzela T. 2016. Airway remodeling in chronic obstructive pulmonary disease and asthma: the role of matrix metalloproteinase-9. Arch Immunol Ther Exp (Warsz). 64(1):47–55. 10.1007/s00005-015-0345-y PMC4713715 26123447

[CIT0011] Hackett TL, Shaheen F, Zhou S, Wright JL, Churg A. 2014. Fibroblast signal transducer and activator of transcription 4 drives cigarette smoke-induced airway fibrosis. Am J Respir Cell Mol Biol. 51(6):830–839. 24922586 10.1165/rcmb.2013-0369OC

[CIT0012] Huang X, Tan H, Chen B, Deng C. 2010. [Influence of astragalosides and *Panax notoginseng* saponins compatibility on MMP-9 and TIMP-1 after cerebral ischemia-reperfusion in mice]. Zhongguo Zhong Yao Za Zhi. 35:2187–2191. 21046759

[CIT0013] Kawayama T, Kinoshita T, Matsunaga K, Kobayashi A, Hayamizu T, Johnson M, Hoshino T. 2016. Responsiveness of blood and sputum inflammatory cells in Japanese COPD patients, non-COPD smoking controls, and non-COPD nonsmoking controls. Int J Chron Obstruct Pulmon Dis. 11:295–303. 26929615 10.2147/COPD.S95686 PMC4755695

[CIT0014] Kim SW, Kim ES, Moon CM, Kim TI, Kim WH, Cheon JH. 2012. Abnormal genetic and epigenetic changes in signal transducer and activator of transcription 4 in the pathogenesis of inflammatory bowel diseases. Dig Dis Sci. 57(10):2600–2607. 22569826 10.1007/s10620-012-2199-z

[CIT0015] Li H, Yang T, Li FY, Ning Q, Sun ZM. 2016. TLR4 Overexpression inhibits endothelial pas domain-containing protein 1 expression in the lower respiratory tract of patients with chronic COPD. Cell Physiol Biochem. 39(2):685–692. 27442627 10.1159/000445659

[CIT0016] Li SY, Li JS, Wang MH, Xie Y, Yu XQ, Sun ZK, Ma LJ, Zhang W, Zhang HL, Cao F, Pan YC. 2012. Effects of comprehensive therapy based on traditional Chinese medicine patterns in stable chronic obstructive pulmonary disease: a four-center, open-label, randomized, controlled study. BMC Complement Altern Med. 12:197–197. 23107470 10.1186/1472-6882-12-197 PMC3528455

[CIT0017] Li Y, Li SY, Li JS, Deng L, Tian YG, Jiang SL, Wang Y, Wang YY. 2012. A rat model for stable chronic obstructive pulmonary disease induced by cigarette smoke inhalation and repetitive bacterial infection. Biol Pharm Bull. 35(10):1752–1760. 22863994 10.1248/bpb.b12-00407

[CIT0018] Li Y, Lu Y, Zhao Z, Wang J, Li J, Wang W, Li S, Song L. 2016. Relationships of MMP-9 and TIMP-1 proteins with chronic obstructive pulmonary disease risk: A systematic review and meta-analysis. J Res Med Sci. 21:12. 27904558 10.4103/1735-1995.178737 PMC5122186

[CIT0019] Mullings RE, Wilson SJ, Puddicombe SM, Lordan JL, Bucchieri F, Djukanovic R, Howarth PH, Harper S, Holgate ST, Davies DE. 2001. Signal transducer and activator of transcription 6 (STAT-6) expression and function in asthmatic bronchial epithelium. J Allergy Clin Immunol. 108(5):832–838. 11692112 10.1067/mai.2001.119554

[CIT0020] Osuka S, Hashimoto N, Sakamoto K, Wakai K, Yokoi K, Hasegawa Y. 2015. Risk stratification by the lower limit of normal of FEV1/FVC for postoperative outcomes in patients with COPD undergoing thoracic surgery. Respir Investig. 53(3):117–123. 10.1016/j.resinv.2015.01.005 25951098

[CIT0021] Page BD, Croucher DC, Li ZH, Haftchenary S, Jimenez-Zepeda VH, Atkinson J, Spagnuolo PA, Wong YL, Colaguori R, Lewis AM, et al. 2013. Inhibiting aberrant signal transducer and activator of transcription protein activation with tetrapodal, small molecule Src homology 2 domain binders: promising agents against multiple myeloma. J Med Chem. 56(18):7190–7200. 23968501 10.1021/jm3017255

[CIT0022] Raherison C. 2011. [Epidemiology of chronic obstructive pulmonary disease]. Rev Prat. 61:769–773. 21826918

[CIT0023] Shi Q, Liu Z, Yang Y, Geng P, Zhu YY, Zhang Q, Bai F, Bai G. 2009. Identification of anti-asthmatic compounds in *Pericarpium citri* reticulatae and evaluation of their synergistic effects. Acta Pharmacol Sin. 30(5):567–575. 19363516 10.1038/aps.2009.36 PMC4002823

[CIT0024] Wang C, Ding H, Tang X, Li Z, Gan L. 2017a. Effect of liuweibuqi capsules in pulmonary alveolar epithelial cells and COPD through JAK/STAT pathway. Cell Physiol Biochem. 43(2):743–756. 28950251 10.1159/000481558

[CIT0025] Wang C, Ding H, Tang X, Li Z, Gan L. 2017b. Effect of Liuweibuqi capsules on the balance between MMP-9 and TIMP1 and viability of alveolar macrophages in COPD. Biosci Rep. 37. DOI:10.1042/BSR20170880 PMC5603752 28831024

[CIT0026] Wang C, Li Z, Liu X, Peng Q, Li F, Li D, Wang C. 2015. Effect of Liuweibuqi capsule, a Chinese patent medicine, on the JAK1/STAT3 pathway and MMP9/TIMP1 in a chronic obstructive pulmonary disease rat model. J Tradit Chin Med. 35(1):54–62. 25842729 10.1016/s0254-6272(15)30009-1

[CIT0027] Wu L, Chen Y, Xu Y, Guo X, Li X, Zhang AL, May BH, Xue CC, Wen Z, Lin L. 2013. Oral huangqi formulae for stable chronic obstructive pulmonary disease: a systematic review and meta-analysis. Evid Based Complement Alternat Med. 2013:705315. 23606889 10.1155/2013/705315 PMC3623121

[CIT0028] Wu X, Feng X, He Y, Gao Y, Yang S, Shao Z, Yang C, Wang H, Ye Z. 2016. IL-4 administration exerts preventive effects via suppression of underlying inflammation and TNF-α-induced apoptosis in steroid-induced osteonecrosis. Osteoporos Int. 27(5):1827–1837. 26753542 10.1007/s00198-015-3474-6

[CIT0029] Yao H, Hwang JW, Sundar IK, Friedman AE, McBurney MW, Guarente L, Gu W, Kinnula VL, Rahman I. 2013. SIRT1 redresses the imbalance of tissue inhibitor of matrix metalloproteinase-1 and matrix metalloproteinase-9 in the development of mouse emphysema and human COPD. Am J Physiol Lung Cell Mol Physiol. 305(9):L615–624. 24039251 10.1152/ajplung.00249.2012 PMC3840276

[CIT0030] Zizzo G, Cohen PL. 2013. IL-17 stimulates differentiation of human anti-inflammatory macrophages and phagocytosis of apoptotic neutrophils in response to IL-10 and glucocorticoids. J Immunol. 190(10):5237–5246. 23596310 10.4049/jimmunol.1203017 PMC3677729

